# ZrO_2_ Ferroelectric Field-Effect Transistors Enabled by the Switchable Oxygen Vacancy Dipoles

**DOI:** 10.1186/s11671-020-03353-6

**Published:** 2020-05-24

**Authors:** Huan Liu, Yue Peng, Genquan Han, Yan Liu, Ni Zhong, Chungang Duan, Yue Hao

**Affiliations:** 1 State Key Discipline Laboratory of Wide Band Gap Semiconductor Technology, School of Microelectronics, Xidian University, Xi’an, 710071 China; 2 Key Laboratory of Polar Materials and Devices, Ministry of Education, East China Normal University, Shanghai, China

**Keywords:** FeFET, ZrO_2_, Memory, Germanium, Amorphous

## Abstract

This paper investigates the impacts of post-rapid thermal anneal (RTA) and thickness of ZrO_2_ on the polarization *P* and electrical characteristics of TaN/ZrO_2_/Ge capacitors and FeFETs, respectively. After the RTA ranging from 350 to 500 °C, TaN/ZrO_2_/Ge capacitors with 2.5 and 4 nm-thick amorphous ZrO_2_ film exhibit the stable *P*. It is proposed that the ferroelectric behavior originates from the migration of the voltage-driven dipoles formed by the oxygen vacancies and negative charges. FeFETs with 2.5 nm, 4 nm, and 9 nm ZrO_2_ demonstrate the decent memory window (MW) with 100 ns program/erase pulses. A 4-nm-thick ZrO_2_ FeFET has significantly improved fatigue and retention characteristics compared to devices with 2.5 nm and 9 nm ZrO_2_. The retention performance of the ZrO_2_ FeFET can be improved with the increase of the RTA temperature. An MW of ~ 0.46 V is extrapolated to be maintained over 10 years for the device with 4 nm ZrO_2_.

## Background

Doped poly-HfO_2_ ferroelectric field-effect transistors (FeFETs) have attracted considerable interest in the non-volatile memory (NVM) applications due to their CMOS process compatibility [1]. Although the decent electrical performance has been demonstrated in doped HfO_2_-based FeFETs [2], some fundamental limitations still plague their practical applications, including the high thermal budget of 500 °C annealing required to form orthorhombic crystal phases and the undesired leakage current along the grain boundaries with the scaling down of ferroelectric thickness. Ferroelectricity has been widely observed in a variety of different materials, e.g., Sb_2_S_3_ nanowires [3], GaFeO_3_ film [4], LaAlO_3_-SrTiO_3_ film [5], and amorphous Al_2_O_3_ containing nanocrystals [6, 7]. Recently, we reported the FeFETs with partially crystallized ZrO_2_ gate insulator functioning as NVM and analog synapse [8]. Although the ZrO_2_ transistors exhibited decent electrical performance with the thinner thickness compared to the reported doped HfO_2_, the underlying mechanism for the ferroelectricity in ZrO_2_ film remains unclear. It is critical and important to elucidate the origin of the switchable polarization *P* for evaluating the performance limit of ZrO_2_ FeFETs.

In this work, TaN/ZrO_2_/Ge FeFETs with 2.5 nm, 4 nm, and 9 nm-thick insulators are fabricated. The switchable *P* in TaN/ZrO_2_/Ge capacitor is proposed to originate from the migration of voltage-driven oxygen vacancies and negative charges. The impacts of ZrO_2_ thickness and the post-rapid thermal annealing (RTA) on the *P* of TaN/ZrO_2_/Ge and the memory window (MW), endurance, and retention characteristics of FeFETs are investigated.

## Methods

FeFETs with ZrO_2_ gate insulator were fabricated on 4-in. n-Ge(001) substrate using a similar process in [8, 9]. After the pre-gate cleaning in the diluted HF (1:50) solution, Ge wafers were loaded into an atomic layer deposition (ALD) chamber. ZrO_2_ films with thicknesses of 2.5 nm, 4 nm, and 9 nm were deposited at 250 °C using TDMAZr and H_2_O as precursors of Zr and O, respectively. A 100-nm-thick TaN gate electrode was deposited by reactive sputtering. After the gate electrode formation, the source/drain (S/D) regions were implanted by BF_2_^+^ at a dose of 1 × 10^15^ cm^−2^ and an energy of 20 keV. A total of 15 nm nickel (Ni) S/D contacts were formed by a lift-off process. Finally, the RTA at 350, 450, and 500 °C for 30 s was carried out.

**Fig. 1:**
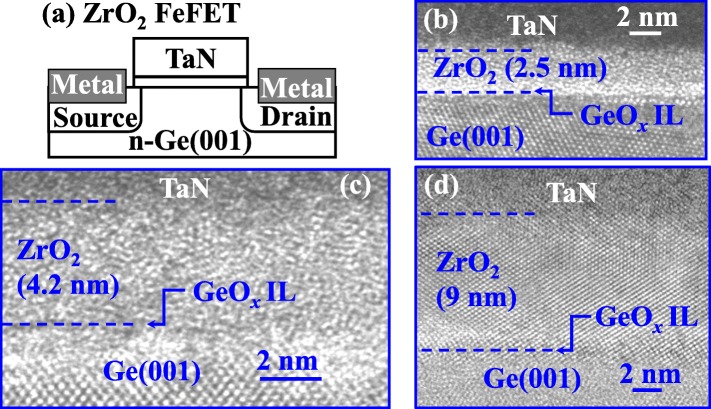
**a** Schematic of the fabricated TaN/ZrO_2_/Ge FeFET. **b**, **c**, and **d** HRTEM images of the TaN/ZrO_2_/Ge stacks with different ZrO_2_ thicknesses. The samples underwent an RTA at 500 °C for 30 s

Figure 1 a shows the schematic of the fabricated transistor. Figure 1b–d shows the transmission electron microscope (TEM) images of the TaN/ZrO_2_/Ge samples with 2.5, 4, and 9 nm-thick ZrO_2_, respectively. All the samples underwent an RTA at 500 °C for 30 s. The 2.5 nm ZrO_2_ sample remains an insulator film after the annealing. For the 4 nm sample, although some nanocrystals are observed, ZrO_2_ maintains to be an amorphous layer. While full crystallization occurs for the 9 nm ZrO_2_ film. Notably, an interfacial layer (IL) of GeO_*x*_ exists between the ZrO_2_ and Ge channel region, although it is too thin to be observed in the TEM images.

## Results and Discussion

**Fig. 2:**
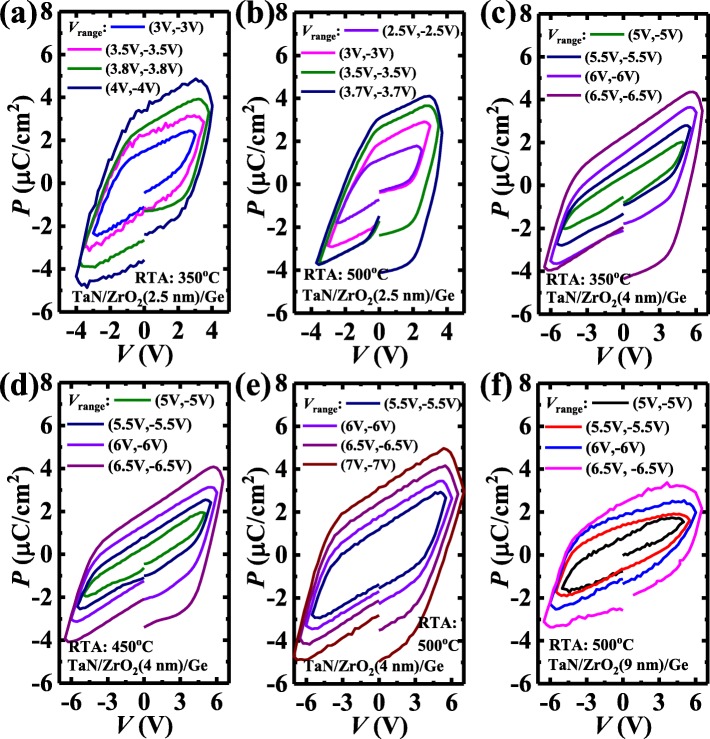
Measured *P* vs. *V* characteristics of the TaN/ZrO_2_/Ge capacitors with different ZrO_2_ thicknesses and various annealing temperatures

**Fig. 3:**
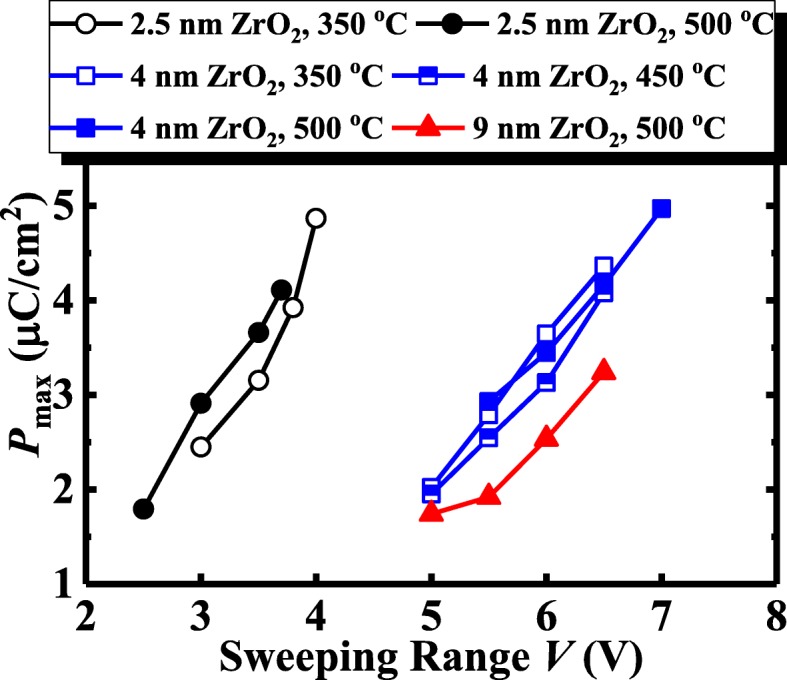
Comparison of *P*_max_ as a function of *V*_range_ for the TaN/ZrO_2_/Ge capacitors with different ZrO_2_ thicknesses and various annealing temperatures

Figure 2 shows the *P* vs. voltage (*V*) curves for the TaN/ZrO_2_/Ge capacitors with different ZrO_2_ thicknesses and different annealing temperatures. The solid lines with different colors represent the minor loops with various sweeping voltage range (*V*_range_). The measurement frequency is 1 kHz. The 2.5 nm and 4 nm ZrO_2_ devices can exhibit stable ferroelectricity after an RTA at 350 °C. Figure 3 plots the remnant *P* (*P*_r_) as a function of the sweeping *V* range curves for the capacitors annealed at various temperatures.

Figure 3 shows the comparison of *P*_max_ as a function of *V*_range_ for the TaN/ZrO_2_/Ge capacitors with the different ZrO_2_ thicknesses and the various RTA temperatures. For the 4 nm ZrO_2_ devices, as the annealing temperature increases from 350 to 450 °C, a larger *V*_range_ is required to obtain a fixed *P*_max_. This is attributed to the fact that the higher annealing temperature produces the thicker interfacial layers (ILs) between at Ge/ZrO_2_ and ZrO_2_/TaN interfaces, leading to a larger unified capacitance equivalent thickness (CET). For the 2.5 nm ZrO_2_ capacitors, the sample with 500 °C annealing has a lower *V*_range_ than does the 350 °C annealing sample with the same *P*_max_. Although the ILs get thicker with the increased RTA temperature, some ZrO_2_ was consumed by the oxygen scavenging and interdiffusion at the interface. For the very thin ZrO_2_ device, the latter is dominant. Compared to the 2.5 nm ZrO_2_ capacitor, a much larger *V*_range_ is required to achieve a similar *P*_max_. However, the 9 nm ZrO_2_ capacitor does not exhibit the higher *V*_range_ in comparison with the 4 nm device. This is due to the crystal ZrO_2_ that has a much higher *κ* value than does the amorphous film, which significantly reduces the CET of the 9 nm device.

**Fig. 4:**
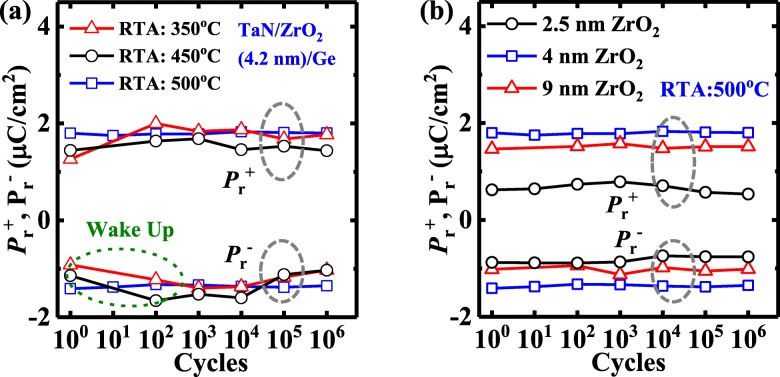
**a***P*_r_ vs. the number of ms-pulse sweeping cycles for 4 nm ZrO_2_ capacitors with different RTA temperatures. **b***P*_r_ vs. number of ms-pulse sweeping cycles for the ZrO_2_ capacitors after annealing at 500 °C

Figure 4a shows the extracted evolution of the positive and negative *P*_r_, denoted by ${P}_{\mathrm{r}}^{+}$and ${P}_{\mathrm{r}}^{-}$, respectively, for the 4 nm-thick ZrO_2_ capacitors with RTA at different temperatures over 10^6^ sweeping cycles measured at 1 kHz. Devices annealed at 350 °C and 450 °C exhibit the obvious wake-up effect. No wake up or imprint is observed for the 4 nm ZrO_2_ ferroelectric capacitor underwent annealing at 500 °C. Figure 4b compares the *P*_r_ as a function of sweeping cycles for the devices with the different ZrO_2_ thicknesses. The 4 nm ZrO_2_ ferroelectric capacitor achieves improved stability of *P*_r_ endurance compared to the 2.5 nm and 9 nm devices during the 10^6^ endurance test.

**Fig. 5:**
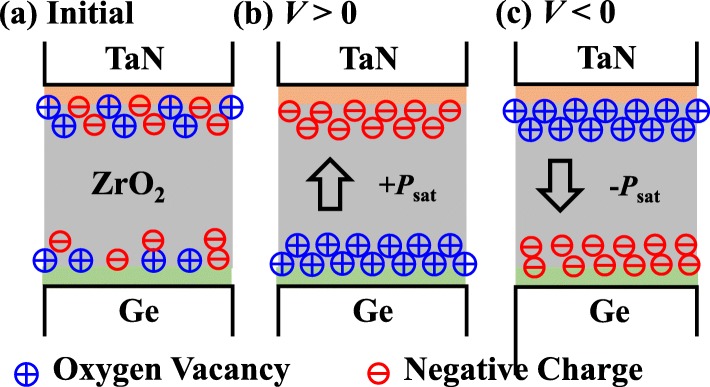
Schematics of the mechanism for switchable *P* in ZrO_2_ capacitors, which is attributed to the migration of voltage-driven oxygen vacancies and negative charges to form dipoles

The switching *P* is observed in amorphous ZrO_2_ capacitance, and it is inferred that the mechanism must be different from that of the reported doped poly-HfO_2_ ferroelectric films. We propose that the underlying mechanism for ferroelectric behavior is related to the oxygen vacancy dipoles. It is well known that, as TaN metal deposited, the Ta oxygen scavenger layers will increase the oxygen vacancy concentration inside ZrO_2_ [10]. Oxygen vacancies also appear at the ZrO_2_/Ge interface. Figure 5 shows the schematics of the switchable *P* in TaN/ZrO_2_/Ge originating from the migration of oxygen vacancies and negative charges to form the positive and negative dipoles. It is speculated that the negative charges in ZrO_2_ are related to the Zr vacancy [11], which is similar to those in Al_2_O_3_ film [12]. The migration of the voltage-driven oxygen vacancies has been widely demonstrated in resistive random-access memory devices [13, 14]. Notably, this is the first demonstration of three-terminal non-volatile transistors dominated by the voltage-driven oxygen vacancies.

**Fig. 6:**
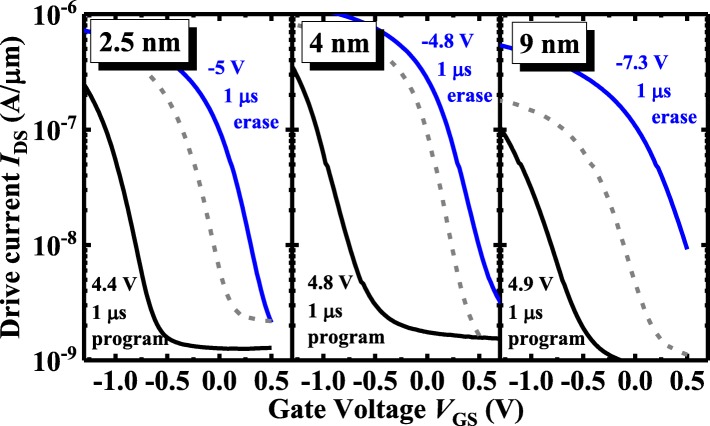
Measured *I*_DS_-*V*_GS_ curves of the 2.5, 4, and 9 nm-thick ZrO_2_ FeFETs for the initial and two polarization states with 1 μs P/E pulses

The *P-V* hysteresis enables the ZrO_2_ FeFETs to obtain a large and stable MW for the embedded NVM (eNVM) applications. Figure 6 shows the measured *I*_DS_-*V*_GS_ curves of 2.5, 4, and 9 nm ZrO_2_ FeFETs for the two polarization states with 1 μs program/erase (P/E) conditions. The transistors were annealed at 500 °C. Program (erase) operation is achieved by applying positive (negative) voltage pulses to the gate of the ZrO_2_ FeFETs, to raise (lower) its threshold voltage (*V*_TH_). *V*_TH_ is defined as *V*_GS_ at 100 nA·W/L, and MW is defined as the maximum change in *V*_TH_. All the FeFETs with various ZrO_2_ thicknesses have the MW above 1 V with 1 μs P/E pulses. To achieve a similar MW, a higher erase voltage is needed for the 9 nm ZrO_2_ FeFET compared to the other two transistors. It is seen that a larger magnitude erase *V*_GS_ is required to obtain the roughly equal shift of *I-V* relative to the initial curve compared to the program *V*_GS_. It is speculated that the oxygen vacancies contributing to the *P* mainly come from the reaction between TaN and ZrO_2_, like the initial state of the device in Fig. 5a. As a positive *V*_GS_ (program) is applied, the oxygen vacancies diffuse and accumulate in the layer near the ZrO_2_/Ge interface (Fig. 5b), where the distribution of the oxygen vacancy dipoles is quite different from the initial state. So it is easy to shift the *I-V* curve to a higher |*V*_TH_| with a positive *V*_GS_. However, as a negative *V*_GS_ (erase) is applied, the back diffusion of oxygen vacancies brings the gate stack back to its original state (Fig. 5c). So the magnitude of the negative erase *V*_GS_ has to be increased to achieve the equivalent shift of *I-V* to the positive program *V*_GS_.

**Fig. 7:**
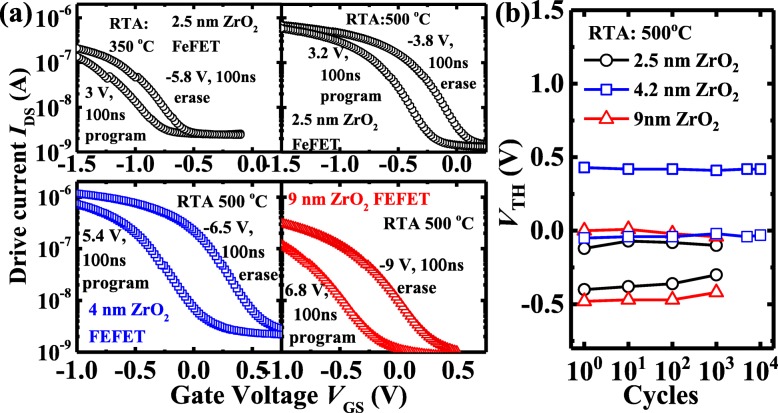
**a***I*_DS_-*V*_GS_ curves of the 2.5, 4, and 9 nm-thick ZrO_2_ FeFETs for the two polarization states with 100 ns P/E pulses. The devices underwent an RTA at 500 °C. **b** FeFET with 4 nm ZrO_2_ has an improved endurance compared to the 2.5 and 9 nm ZrO_2_ transistors

As the P/E pulse width is reduced to 100 ns, the ZrO_2_ FeFETs still demonstrate the decent MW, as shown in Fig. 7a. Especially, the transistor with 2.5 nm ZrO_2_ annealed at 350 °C achieves an MW of 0.28 V. Figure 7b plots MW vs. cycle number for the FeFETs with various ZrO_2_ thicknesses with 100 ns P/E pulse condition. The 4 nm ZrO_2_ device achieves a significantly improved endurance performance compared to the 2.5 nm and 9 nm ZrO_2_ FeFETs, which exhibit the obvious wake-up effect and fatigue within 10^3^ cycles.

**Fig. 8:**
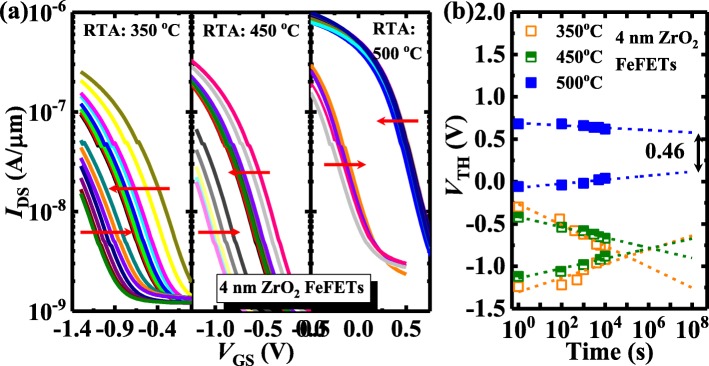
**a** The evolution of *I*_DS_-*V*_GS_ curves for the two polarization states of the 4 nm ZrO_2_ FeFETs with different RTA temperatures. **b** The 4 nm ZrO_2_ device annealed at 500 °C has a much better retention performance compared to the transistors with RTA at the lower temperatures

**Fig. 9:**
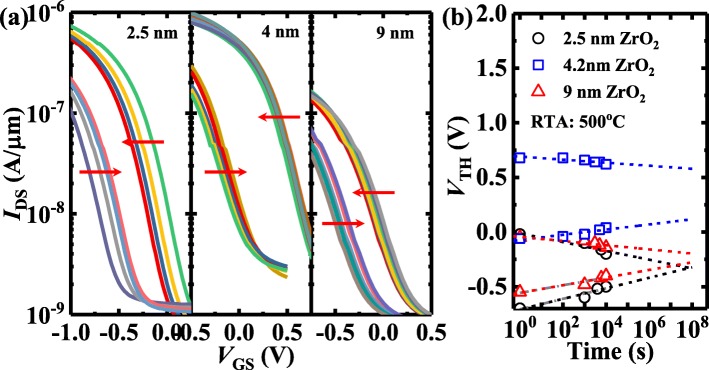
**a** The evolution of *I*_DS_-*V*_GS_ curves for the two polarization states for the 2.5, 4, and 9 nm-thick ZrO_2_ FeFETs underwent a RTA at 500 °C. **b** The 4 nm ZrO_2_ device has an improved retention performance compared to the transistors with 2.5 and 9 nm-thick ZrO_2_

Finally, the retention testing of the ZrO_2_ FeFETs is characterized and shown in Figs. 8 and 9. Figure 8 a shows the evolution of *I*_DS_-*V*_GS_ curves for the two polarization states of the 4 nm ZrO_2_ FeFETs underwent RTA at 350, 450, and 500 °C. The charge trapping leads to the reduction of the devices with the time. As shown in Fig. 8b, the retention performance of the devices can be improved with the increase of the RTA temperature. An MW of ~ 0.46 V is extrapolated to be maintained over 10 years. Figure 9 compares the retention characteristics of the FeFETs with different ZrO_2_ thicknesses. The 4 nm ZrO_2_ device has an improved retention performance compared to the transistors with 2.5 and 9 nm-thick ZrO_2_.

## Conclusions

In summary, amorphous ZrO_2_ ferroelectric capacitors are experimentally demonstrated, and the ferroelectricity is speculated to be due to the migration of the voltage-driven dipoles formed by the oxygen vacancies and negative charges. FeFETs with 2.5 nm, 4 nm, and 9 nm ZrO_2_ have the MW above 1 V with 1 μs P/E pulses. The improved fatigue and retention characteristics are obtained in the 4 nm-thick ZrO_2_ FeFET in comparison with the devices with 2.5 nm and 9 nm ZrO_2_. The retention test indicates that the 4 nm ZrO_2_ transistor keeps an extrapolated 10-year MW of ~ 0.46 V.

## Data Availability

The datasets supporting the conclusions of this article are included in the article.

## Abbreviations

RTA: Rapid thermal anneal
IL: Interfacial layer
TaN: Tantalum nitride
FeFET: Ferroelectric field-effect transistors
TDMAZr: Tetrakis (dimethylamido) zirconium
Ge: Germanium
ZrO_2_: Zirconium dioxide
ALD: Atomic layer deposition
HF: Hydrofluoric acid
BF_2_^+^: Boron fluoride ion
MW: Memory window
NVM: Non-volatile memory
*P*_r_: Remnant polarization
TEM: Transmission electron microscope
Ni: Nickel
*P*_max_: Maximum polarization
RTA: Repaid thermal annealing
*V*_range_: Sweeping voltage range
